# Autophagic Degradation of Gasdermin D Protects against Nucleus Pulposus Cell Pyroptosis and Retards Intervertebral Disc Degeneration In Vivo

**DOI:** 10.1155/2021/5584447

**Published:** 2021-06-17

**Authors:** Zhiwei Liao, Suyun Li, Rong Liu, Xiaobo Feng, Yunsong Shi, Kun Wang, Shuai Li, Yukun Zhang, Xinghuo Wu, Cao Yang

**Affiliations:** ^1^Department of Orthopaedics, Union Hospital, Tongji Medical College, Huazhong University of Science and Technology, Wuhan 430022, China; ^2^Department of Orthopaedic Surgery, Puren Hospital of Wuhan, Wuhan University of Science and Technology, Wuhan, China

## Abstract

Intervertebral disc degeneration (IDD) is the primary culprit of low back pain and renders heavy social burden worldwide. Pyroptosis is a newly discovered form of programmed cell death, which is also involved in nucleus pulposus (NP) cells during IDD progression. Moderate autophagy activity is critical for NP cell survival, but its relationship with pyroptosis remains unknown. This study is aimed at investigating the relationship between autophagy and pyroptotic cell death. The pyroptosis executor N-terminal domain of gasdermin D (GSDMD-N) and inflammation-related proteins were measured in lipopolysaccharide- (LPS-) treated human NP cells. Inhibition of autophagy by siRNA transfection and chemical drugs aggravated human NP cell pyroptosis. Importantly, we found that the autophagy-lysosome pathway and not the proteasome pathway mediated the degradation of GSDMD-N as lysosome dysfunction promoted the accumulation of cytoplasmic GSDMD-N. Besides, P62/SQSTM1 colocalized with GSDMD-N and mediated its degradation. The administration of the caspase-1 inhibitor VX-765 could reduce cell pyroptosis as confirmed in a rat disc IDD model *in vivo*, whereas ATG5 knockdown significantly accelerated the progression of IDD. In conclusion, our study indicated that autophagy protects against LPS-induced human NP cell pyroptosis via a P62/SQSTM1-mediated degradation mechanism and the inhibition of pyroptosis retards IDD progression *in vivo*. These findings deepen the understanding of IDD pathogenesis and hold implications in unraveling therapeutic targets for IDD treatment.

## 1. Introduction

Intervertebral disc degeneration (IDD) is considered as the primary pathological basis for painful spine diseases. Intervertebral disc (IVD) tissue mainly serves as an intervertebral junction and buffers mechanical pressure. This sandwich-like organ is composed of outer annulus fibrosus, inner nucleus pulposus (NP), and cartilaginous endplates at ends [1]. IDD is a common musculoskeletal degenerative disease characterized by chronic inflammation and progressive cell death [2, 3]. The death of resident NP cells accompanied by extracellular matrix metabolism disorder is closely related to IDD progression [4]. Different cell death pathways (i.e., programmed cell death) could be involved in IDD and interact with each other to accelerate IDD progression [1]. Therefore, further investigation about the mechanisms of NP cell death is required and it may provide potential therapeutic targets for IDD treatment.

Pyroptosis is a form of regulated cell death initiated by inflammasomes and caspase activation, which induces pore formation on the plasma membrane and cell swelling or lysis [5]. Specifically, activation of inflammatory caspases induces the cleavage of gasdermin D (GSDMD), releasing the N-terminal domain (GSDMD-N), which leads to cell membrane perforation and lytic death [6, 7]. The inflammasome-mediated cell pyroptosis plays a critical role in the pathogenesis of various diseases, including cancer, cardiovascular diseases, and osteoarthritis [8–10]. Previous studies have indicated that the sustained activation of inflammasomes exists during the progression of IDD [11, 12]; however, the role of NP cell pyroptosis in the pathogenesis of IDD remains unclear.

Autophagy is a conserved cellular mechanism that assists cells to adapt and protect themselves in response to stress [13]. Autophagy contributes to cellular quality control, including removing damaged proteins and organelles, which is closely associated with various forms of cell death [14, 15]. Several studies showed that autophagy limits the activation of inflammasomes and alleviates the secretion of inflammatory cytokines [16, 17]. It is reasonable to assume that autophagy regulates the activation of inflammasome and then affects the outcome of cell pyroptosis. However, the relationship between cell pyroptosis and autophagy is complicated. Based on our previous studies, autophagy activation protected NP cell against stress-induced cell death, whereas autophagy inhibition promotes cell apoptosis [18, 19]. Therefore, we assumed that reasonable autophagy activity is beneficial for NP cell survival and may play a role in regulating inflammasome activation and NP cell pyroptosis. The underlying mechanism of autophagy on regulating cell pyroptosis still needs to be investigated.

To test our hypothesis and elucidate the underlying mechanisms, we assessed the relationship between GSDMD-N and autophagy activation based on the detection of NP tissue specimens. We further treated NP cells with lipopolysaccharide (LPS) to establish the cell pyroptosis model and investigated the effects of autophagy intervention on NP cells *in vitro*, as well as in a puncture-induced rat IDD model *in vivo*. Our study revealed that autophagy inhibits the GSDMD-related NP cell pyroptosis and blocks GSDMD-N release via a P62-mediated degradative mechanism. Accordingly, this study helps to better understand the relationship between pyroptosis and autophagy during IDD and may provide a prospective strategy for IDD therapy.

## 2. Methods and Materials

### 2.1. Tissue Collection and Ethic Statement

Human NP tissues were obtained from patients that underwent intervertebral fusion surgery due to lumbar fracture or degenerative disc diseases. According to the magnetic resonance (MRI) images of patients, the IDD degree was assessed based on the Pfirrmann MRI-grade system [20]. Usually, Pfirrmann Grade I or II was considered as nondegenerative IVD (NC), and Pfirrmann Grade III, IV, or V belongs to degenerative discs (IDD). The tissue specimens were fixed in 4% formaldehyde and used for histological analysis or frozen stored for protein and RNA analysis. All experimental protocols including medical records, NP tissue collection, and cell interventions were approved by the Ethics Committee of Tongji Medical College, Huazhong University of Science and Technology.

### 2.2. Cell Culture

NP cells were isolated and cultured as previously described [21]. Briefly, NP tissues were cut into pieces and enzymatically digested in 0.2% type II collagenase. After filtering and washing with phosphate-buffered saline (PBS), the suspension was centrifuged to collect the sediment. The isolated cells in precipitation were cultured in Dulbecco's modified Eagle medium containing 15% fetal bovine serum (Gibco, USA) and 1% penicillin/streptomycin. The culture medium was replaced twice a week, and cells from the second passage were prepared for use.

### 2.3. Western Blot

Cell lysates or milled tissues were treated with RIPA lysis buffer (Beyotime, China), and proteins were extracted. The proteins were separated by polyacrylamide gel electrophoresis (SDS-PAGE) and then transferred onto PVDF membranes. The membranes were blocked with 5% skim milk. The primary antibodies used were GSDMD (Proteintech, 1 : 5000), GSDMD-N (CST, 1 : 1000), caspase-1 (Proteintech, 1 : 1000), NLRP3 (Abcam, 1 : 1000), LC-3 (CST, 1 : 1000), Beclin-1 (Boster,1 : 1000), P62 (Boster, 1 : 1000), COL2A1 (Proteintech, 1 : 6000), MMP3 (Proteintech, 1 : 4000), and GAPDH (Abcam, 1 : 10000). After incubation with horseradish peroxidase-conjugated secondary antibodies (Boster, China) and washed with Tris-buffered saline tween (TBST) buffer, the bands were visualized and detected using the enhanced chemiluminescence system. The band intensity value of proteins was calculated using ImageJ 1.52a (National Institutes of Health, USA) and normalized to GAPDH.

### 2.4. Evaluation of Cell Pyroptosis

Pyroptosis of NP cells was evaluated by extracellular lactate dehydrogenase (LDH) level and staining of caspase-1-related cell death. The activity of released LDH was assessed by LDH Activity Assay Kit (Boster, China). To evaluate the double-positive staining of caspase-1 and PI, the Caspase-1 Assay Kit (Immunochemistry Technologies, LCC, USA) was used to detect the caspase-1 activity and cell death. Cell detection and characterization were carried out using the FACSCalibur flow cytometer (BD Biosciences, USA), and data was analyzed by FlowJo X (Tree Star, USA).

### 2.5. Enzyme-Linked Immunosorbent Assay (ELISA)

The NP cell supernatant was collected and centrifuged, and then, the contents of TNF-*α*, IL-1*β*, and IL-6 were analyzed using the corresponding ELISA kit (Elabscience Biotechnology, China) according to the manufacturer's instructions. The experiment was performed in triplicates.

### 2.6. Immunoprecipitation

Cell lysates were treated with 50 mM Tris-HCl, 150 mM NaCl, 1 mM EDTA, and 1% NP-40 with protease inhibitor cocktail (Beyotime, China). The sample (500 *μ*g) at 4°C was added with 10 *μ*L of immunoprecipitation antibody (anti-P62, 1 : 100, Abcam) and incubated for 8 h at 4°C with magnetic beads (MCE, China). Then, the immunoprecipitates were separated by magnetic adsorption conducted with western blot assays.

### 2.7. Immunofluorescence Staining

NP cells were fixed with 4% paraformaldehyde and permeabilized with 0.2% Triton X-100. The slides were washed in PBS and blocked with blocking buffer (Beyotime, China) for 1 h. Then, the slides were incubated with primary antibodies overnight. The primary antibodies used were GSDMD-N (CST, 1 : 100), P62 (CST, 1 : 200), and LC-3 (CST, 1 : 200). Nuclei were stained with DAPI (Beyotime, China) for 5 minutes. Images were captured under a fluorescence microscope (Olympus, BX53; Melville, NY). Fluorescence intensity analysis and colocalization analysis were performed by ImageJ 1.52a.

### 2.8. Transmission Electron Microscopy (TEM)

NP cells were fixed in 2.5% glutaraldehyde for 8 h, then postfixed in 2% osmium tetroxide for 1 h, and stained with 2% uranyl acetate for 1 h. After dehydration in an ascending series of acetone, samples were embedded into Araldite. Samples were cut into ultrathin sections by a slicer (Leica, Germany) and then stained with toluidine blue. The sections were observed under a transmission electron microscope (Tecnai G2 20 TWIN, FEI, USA).

### 2.9. Knockdown Experiments

Knockdown of ATG5 or P62 in NP cells was carried out by transfection with small interfering RNA (siRNA). Target siRNA and scrambled siRNA (si-scr) were synthesized by RiboBio (Guangzhou, China): ATG5-siRNA sequence 5′-GCUAUAUCAGGAUGAGAUATT-3′ and P62-siRNA sequence 5′-GUGUGAAUUUCCUGAAGAATT-3′. The siRNAs were transfected with Lipofectamine 2000 (Invitrogen) in NP cells according to the manufacturer's instructions. Transgenic efficacy in NP cells was detected using quantitative real-time polymerase chain reaction (qRT-PCR) at 48 h after transfection.

### 2.10. Quantitative Real-Time Polymerase Chain Reaction (qRT-PCR)

Total RNA extracted with Trizol reagent (Invitrogen) from NP cells was reverse-transcribed and amplified by qRT-PCR according to the standard protocols. The qRT-PCR was performed to quantify GSDMD, ATG5, or P62 mRNA expression levels. The primer sequences were as follows: Homo GSDMD: forward 5′-GAGCCCAGTGCTCCAGAA-3′, reverse 5′-TTGCATGATCTCCCAGGT-3′; Homo ATG5: forward 5′-AAAGATGTGCTTCGAGATGTGTGGT-3′, reverse 5′-GCAAATAGTATGGTTCTGCTTCCCT-3′; Homo P62: forward 5′-GGCTGATTGAGTCCCTCTCCCAGAT-3′, reverse 5′-CGGCGGGGGATGCTTTGAATACTGG-3′; and Homo GAPDH: forward 5′-TCAAGAAGGTGGTGAAGCAGG-3′, reverse 5′-TCAAAGGTGGAGGAGTGGGT-3′. GAPDH was used as an internal control for normalization.

### 2.11. Animal Experiments

Sprague-Dawley rats (SD, 2-month-old, male) purchased from Experimental Animal Center of Tongji Medical College, Huazhong University of Science and Technology, were used for animal experiments. A model of IDD was established by needle puncture as previously described [20, 22, 23]. Briefly, the rats were anaesthetized with 2% (*w*/*v*) pentobarbital (40 mg/kg). To set the disc degeneration model, the IVD of rats (Co 8/9) was punctured with a 20-gauge needle through the tail skin from the dorsal side. The needle was kept in the disc for 10 s to cause the injury. The length of the needle was predetermined to guarantee the puncture depth at approximately 4 mm. The rats were randomly divided into four groups: control group, the group with the puncture of a 33-gauge needle (Hamilton, Benade, Switzerland) as a sham group; IDD group, the group with a 20-gauge needle puncture to induce disc degeneration and with the injection of PBS (2 *μ*L) using a 33-gauge needle; si-ATG5 group, the group with a 20-gauge needle puncture and with injection of *in vivo* ATG5-siRNA (2 *μ*L, 20 *μ*mol/L, RiboBio, China) using a 33-gauge needle; and VX-765 group, the group with a 20-gauge needle puncture and with VX-765 (25 mg/kg) via oral gavage. The injection procedure was conducted biweekly whereas the oral gavage weekly.

### 2.12. Histological Analysis

The discs were harvested one month after the surgery. These samples were fixed in formaldehyde and then decalcified, dehydrated, and embedded in paraffin. The slides of each disc were stained with hematoxylin-eosin (HE), Alcian blue, and Masson staining. Histological grades of discs were evaluated based on the scoring scale [24]. This scale included 5 categories in assessing disc changes, with 0 points for a normal disc and 15 points for a severely degenerated disc. For immunohistochemistry analysis, the sections were deparaffinized and rehydrated. After blocking with 3% hydrogen peroxide for 10 min and 5% bovine serum albumin for 30 min successively, the sections were incubated with the primary antibodies for 8 h at 4°C. The sections were then incubated with an HRP-conjugated secondary antibody and counterstained with hematoxylin. The images of immunohistochemistry were captured, and the positive cells were analyzed using ImageJ 1.52a.

### 2.13. Statistical Analysis

Data are presented as means ± standard deviation (SD). Student's *t*-test and one-way or two-way analysis of variance (ANOVA) with Tukey's *post hoc* test were performed to assess the differences of changes between groups. Statistical significance (^∗^*P* < 0.05; ^∗∗^*P* < 0.01; ^∗∗∗^*P* < 0.001; *P* > 0.05, ns, no significant difference) was calculated using GraphPad Prism 8 (La Jolla, CA, USA).

## 3. Results

### 3.1. Detection of Autophagy and Pyroptosis Level in Human and Rat Intervertebral Discs

**Figure 1:**
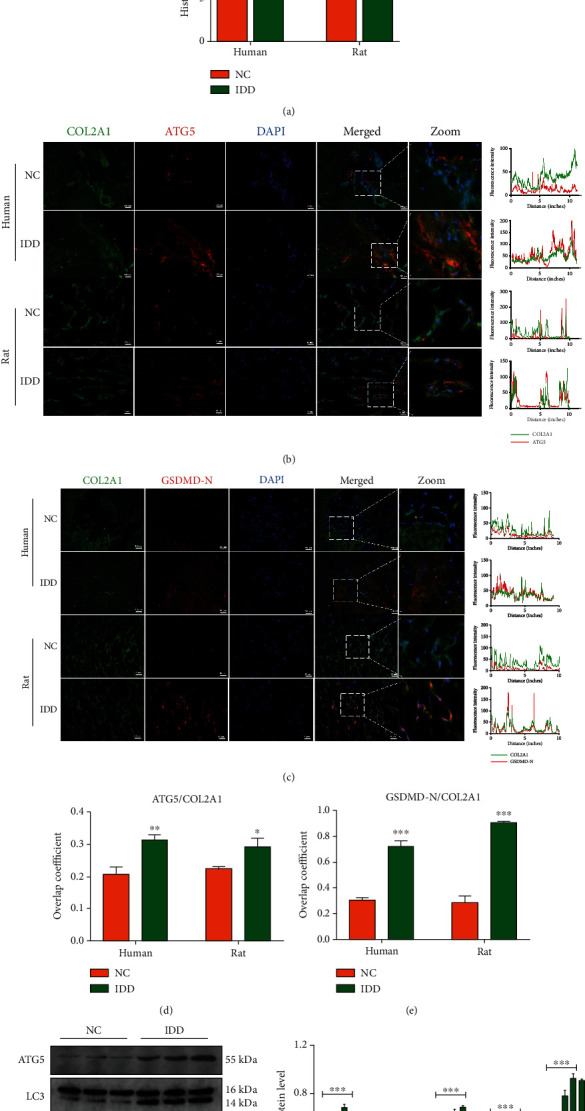
Histological staining results of human and rat intervertebral discs. (a) HE staining showed the morphology of rat discs and human NP tissues (upper panel), and histological grades were calculated based on HE staining (lower panel). (b) Immunofluorescence analysis of ATG5 (red) and type collagen II (green) in rat and human NP tissues. Corresponding fluorescence intensity analysis is listed in the right panel. Scale bar, 50 *μ*m. (c) Immunofluorescence analysis of GSDMD-N (red) and type collagen II (green) in rat and human NP tissues (left panel) and fluorescence intensity results (right panel). Overlap coefficient based on immunofluorescence results showed the colocalization relationship of (d) ATG5 and COL2A1 or (e) GSDMD-N and COL2A1. (f) Western blot analysis of ATG5, LC3, GSDMD-N, COL2A1, and MMP3 in human nondegenerative and degenerative NP tissues and (g) corresponding quantification of protein levels. NC: nondegenerative discs; IDD: degenerative discs. GAPDH was used as an internal control. Data were presented as the means ± SD, *n* = 3. ^∗^*P* < 0.05, ^∗∗^*P* < 0.01, and ^∗∗∗^*P* < 0.001 vs. the NC group.

Histological staining was performed to assess the IDD degree of human and rat normal and degenerative discs. The corresponding histological grades based on tissue morphology and cellularity indicated a higher IDD score in both human and rat IDD groups (Figure 1(a)). The expression levels of autophagy-initiated protein ATG5, the pyroptosis executor GSDMD-N, and the extracellular matrix (ECM) component type II collagen (COL2A1) were detected by immunofluorescence staining (Figures 1(b) and 1(c)). The results showed that higher levels of ATG5 and GSDMD-N were found in degenerative disc tissues (Figures 1(d) and 1(e)). Moreover, protein levels of LC3, ATG5, GSDMD-N, COL2A1, and matrix degradative enzyme, MMP3, were measured by western blot in human IVD tissues (Figure 1(f)). The levels of LC3, ATG5, and GSDMD-N were all increased significantly in the IDD group (Figure 1(g)). These results suggested that autophagy activity and pyroptosis levels were both increased in the degenerative disc tissues.

### 3.2. Evaluation of GSDMD-Mediated Pyroptosis in LPS-Treated NP Cells *In Vitro*

**Figure 2:**
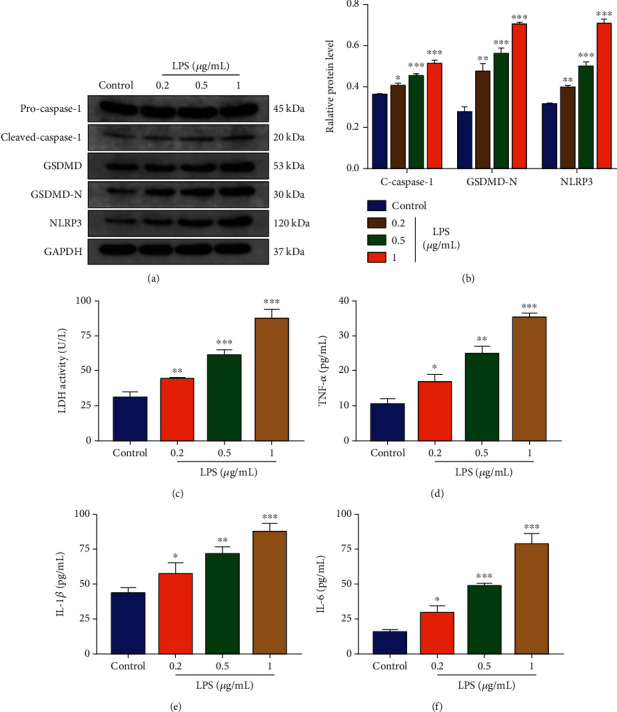
LPS induced NP cell pyroptosis in vitro in a dose-dependent manner. (a) Western blot analysis of pro-caspase-1, cleaved caspase-1, GSDMD, GSDMD-N, and NLRP3 in NP cells treated with LPS (0.2 *μ*g/mL, 0.5 *μ*g/mL, and 1 *μ*g/mL) for 24 h. The control group was treated with equivalent solvent. (b) Quantification of cleaved caspase-1, GSDMD-N, and NLRP3 protein levels. GAPDH was used as an internal control. (c) Measurement of LDH activity in LPS-treated NP cells. ELISA analyzed the levels of secreted (d) TNF-*α*, (e) IL-1*β*, and (f) IL-6 in LPS-treated NP cells. Data were presented as the means ± SD, *n* = 3. ^∗^*P* < 0.05, ^∗∗^*P* < 0.01, and ^∗∗∗^*P* < 0.001 vs. the control group.

NP cells were treated with LPS, a typical pyroptotic phenotype inducer, and related proteins of cell pyroptosis were evaluated (Figure 2(a)). The results indicated that LPS induced the expression of GSDMD-N, cleaved caspase-1 (c-caspase1), and NLRP3 in NP cells in a dose-dependent manner (Figure 2(b)). Besides, it was confirmed that GSDMD-N produced plasma membrane pores and extracellular LDH level was elevated simultaneously [6]. Extracellular LDH measured by an ELISA kit was increased in LPS-treated NP cells (Figure 2(c)). The secretion of inflammatory cytokines TNF-*α*, IL-1*β*, and IL-6 were also increased significantly upon LPS treatment (Figures 2(d)–2(f)). These results showed that LPS promoted the cleavage of GSDMD and induced the pyroptotic phenotype in NP cells in a dose-dependent manner.

### 3.3. Modulation of Autophagy Activity Affects the Outcome of Pyroptotic NP Cell Death

**Figure 3:**
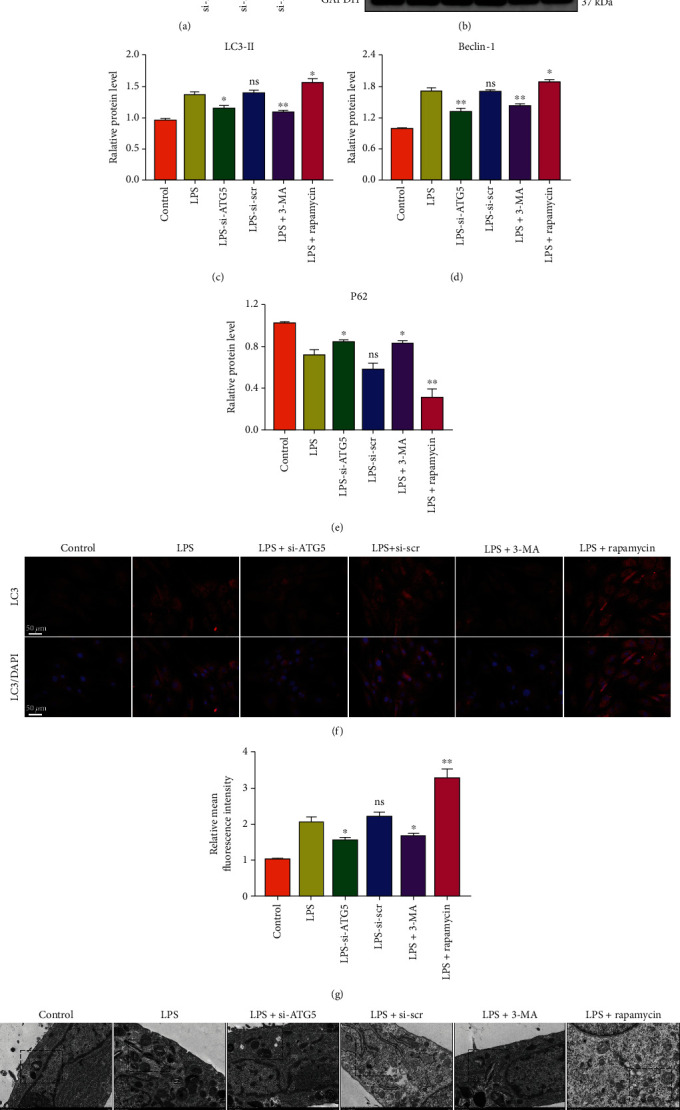
Detection of autophagy activity in NP cells in vitro. (a) The mRNA level of ATG5 in ATG5 siRNA-treated NP cells was analyzed by qRT-PCR. si-ATG5-1 was used in the following experiments. ^∗∗∗^*P* < 0.001 vs. the control group; ns, *P* > 0.05, no significant difference. (b) Western blot analysis of LC3, Beclin-1, and P62. LPS-treated (1 *μ*g/mL, 24 h) NP cells were cotreated with 3-MA (10 mM, 24 h) or rapamycin (1 *μ*M, 24 h). Quantification of (c) LC3-II, (d) Beclin-1, and (e) P62 protein levels. GAPDH was used as an internal control. (f) Immunofluorescence analysis showed the expression level of LC3 and (g) relative mean fluorescence was calculated. Data were presented as the means ± SD, *n* = 3. ^∗^*P* < 0.05 and ^∗∗^*P* < 0.01 vs. the LPS group; ns, *P* > 0.05, no significant difference. (h) TEM images of NP cells indicated the number and morphology of autophagosomes (red arrowheads).

**Figure 4:**
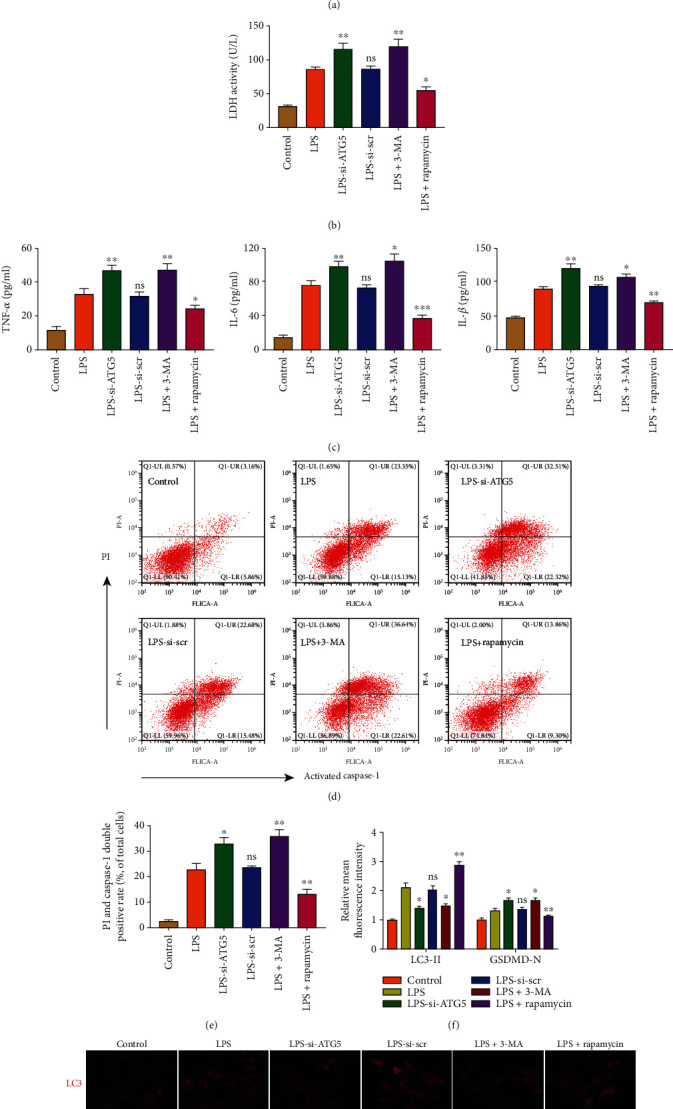
Impaired autophagy activity aggravated LPS-induced NP cell pyroptosis in vitro. (a) Western blot analysis and quantification of NLRP3, cleaved caspase-1, and GSDMD-N protein levels. GAPDH was used as an internal control. (b) Measurement of extracellular LDH activity in NP cells. (c) ELISA analysis assessed the levels of secreted TNF-*α*, IL-1*β*, and IL-6. (d) Flow cytometry analyzed the pyroptotic cells, which were double positive of activated caspase-1 and PI. (e) The rate of double-positive cells was calculated. (g) Double immunofluorescence analysis showed the expression level of LC3 (red) and GSDMD-N (green). Scale bar, 50 *μ*m. (f) The relative mean fluorescence in each group was calculated. Data were presented as the means ± SD, *n* = 3. ^∗^*P* < 0.05, ^∗∗^*P* < 0.01, and ^∗∗∗^*P* < 0.001 vs. the corresponding LPS group; ns, *P* > 0.05, no significant difference.

The effects of autophagy on NP cell pyroptosis were assessed by ATG5 knockdown to inhibit autophagosome formation. The knockdown efficiency was analyzed at the mRNA level (Figure 3(a)). The autophagy inhibitor 3-MA and autophagy inducer rapamycin were used, and the expression levels of autophagy markers LC3-II, Beclin-1, and P62 were assessed by western blotting (Figures 3(b)–3(e)). Immunofluorescence analysis of LC3-II also revealed the number of autophagosomes (Figures 3(f) and 3(g)). TEM images indicated the number and morphology of autophagosomes in different groups (Figure 3(h)). On the other hand, the expression levels of pyroptosis-associated proteins were measured in NP cells (Figure 4(a)). The GSDMD-N, NLRP3, and c-caspase1 expression levels were increased by ATG5 siRNA or 3-MA treatment but reduced by rapamycin treatment. These profiles were consistent with the activity of released LDH and the secretion of inflammatory cytokines (Figures 4(b) and 4(c)). To further assess the NP cell pyroptosis, the cell rates of activated caspase-1 and PI double-positive were analyzed by flow cytometry (Figures 4(d) and 4(e)). Besides, immunofluorescence analysis of GSDMD-N indicated the NP cell pyroptosis in different groups (Figures 4(f) and 4(g)). These results indicated that autophagy inhibition aggravated the LPS-induced NP cell pyroptosis, and autophagy activation ameliorated cell pyroptosis.

### 3.4. Autophagy Mediates NP Cell Pyroptosis via a Degradation Mechanism of GSDMD-N

**Figure 5:**
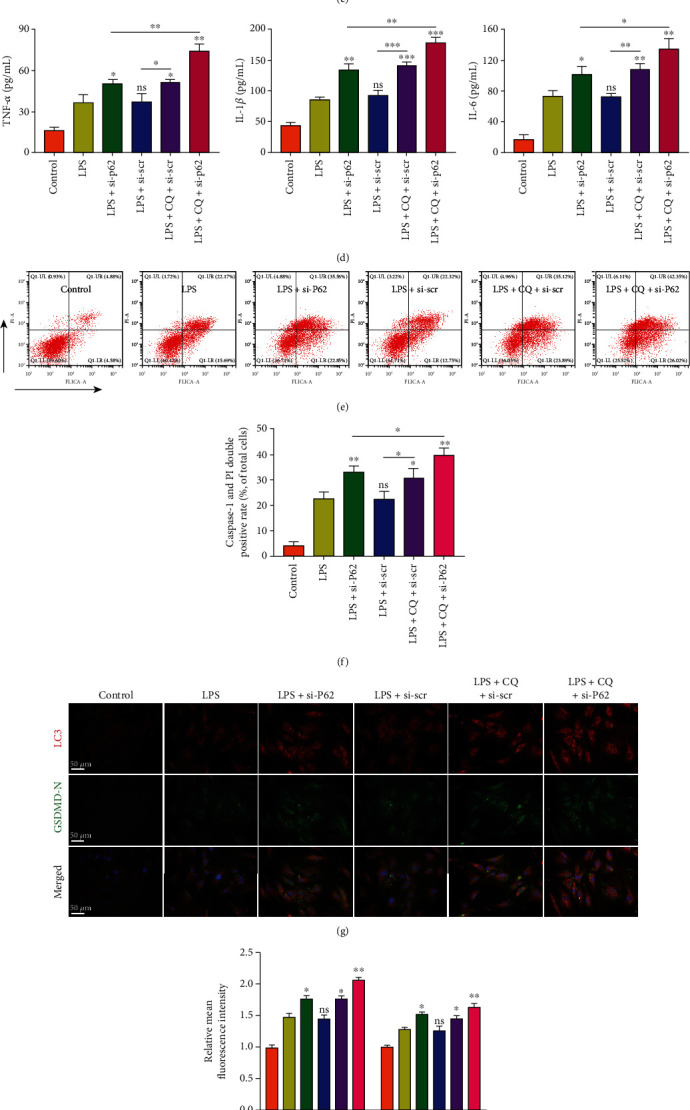
Dysfunction of the autophagy-lysosome pathway promoted the accumulation of cytoplasmic GSDMD-N. (a) The mRNA level of P62 was analyzed by qRT-PCR, and si-P62-3 was selected in the following experiments. ^∗∗^*P* < 0.01 and ^∗∗∗^*P* < 0.001 vs. the control group; ns, *P* > 0.05, no significant difference. (b) Western blot analysis and quantification of P62, NLRP3, cleaved caspase-1, and GSDMD-N. (c) Measurement of extracellular LDH activity. (d) ELISA analyzed the levels of secreted TNF-*α*, IL-1*β*, and IL-6. (e) Flow cytometry analyzed the double-positive cell of activated caspase-1 and PI and (f) calculated the rate of double-positive cells. (g) Immunofluorescence analysis of LC3 (red) and GSDMD-N (green) and (h) the relative mean fluorescence in each group was calculated. (i) Immunofluorescence analysis of P62 (red) and GSDMD-N (green) and (j) the relative mean fluorescence in each group was calculated. Scale bar, 50 *μ*m. Data were presented as the means ± SD, *n* = 3. ^∗^*P* < 0.05, ^∗∗^*P* < 0.01, and ^∗∗∗^*P* < 0.001 vs. the corresponding LPS group; ns, *P* > 0.05, no significant difference.

The autophagy-lysosome degradation pathway is indispensable for controlling cellular protein quality [25]. Cytoplasmic damaged proteins are selected and mediated by specific receptors, such as P62/SQSTM1, which regulates selective degradation via autophagy [26]; however, the role of the autophagy-lysosome pathway in pyroptosis is still unclear. Knockdown of P62 was realized by siRNA, and the efficiency was analyzed accordingly (Figure 5(a)). Chloroquine (CQ) was used to inhibit lysosome function in LPS-treated NP cells. Both si-P62 and CQ treatment increased the expression levels of NLRP3, c-caspase1, and GSDMD-N (Figure 5(b)), results that were in agreement with the activity of released LDH and the secretion of inflammatory cytokines (Figures 5(c) and 5(d)). The rate of pyroptotic cell death was higher in the si-P62 and CQ group than in the control group (Figure 5(e)). Moreover, cotreated si-P62 and CQ in NP cells showed a markedly higher cell pyroptosis rate (Figure 5(f)). Immunofluorescence analysis of LC3 and P62 reflected the dynamic changes of autophagy flux in NP cells (Figures 5(g) and 5(i)). Besides, impaired lysosome function significantly increased the level of cytoplasmic GSDMD-N (Figures 5(h) and 5(j)). These results indicated that autophagy regulated the degradation of GSDMD-N and blockage of the autophagy-lysosome pathway increased the accumulation of cellular GSDMD-N.

### 3.5. P62-Mediated Selective Degradation of GSDMD-N during NP Cell Pyroptosis

**Figure 6:**
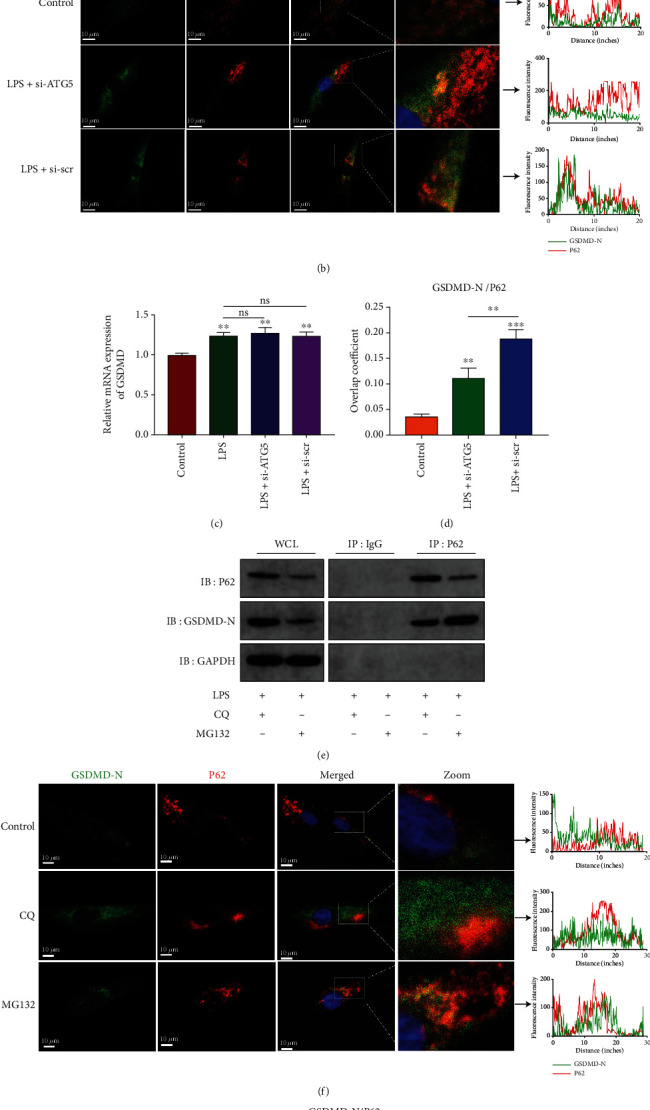
P62 mediated GSDMD-N degradation via an autophagy-lysosome pathway. (a) Immunoprecipitation for P62 was conducted to detect the integration of P62 and GSDMD-N. IgG was as a negative control. WCL: whole cell lysate. (b) Confocal images of GSDMD-N (green) and P62 (red) and fluorescence intensity results (right panel). (c) The mRNA level of GSDMD was analyzed by qRT-PCR. ^∗∗^*P* < 0.01 vs. the control group; ns, *P* > 0.05, no significant difference. (d) Overlap coefficient based on immunofluorescence images showed the colocalization relationship of GSDMD-N and P62. ^∗∗^*P* < 0.01 and ^∗∗∗^*P* < 0.001 vs. the control group. (e) Immunoprecipitation for P62 was conducted to detect the integration of P62 and GSDMD-N in CQ (20 *μ*M, 24 h) or MG132 (10 *μ*M, 24 h) treated NP cells under LPS stimulation. (f) Confocal images of GSDMD-N (green) and P62 (red) and fluorescence intensity measurement (right panel). (g) Overlap coefficient analyzed the colocalization of GSDMD-N and P62. ^∗∗^*P* < 0.01 vs. the CQ group. Data were presented as the means ± SD, *n* = 3.

To further confirm the role of P62/SQSTM1 as an autophagy receptor in GSDMD-N degradation, immunoprecipitation analysis was performed to assess the integration of P62 and GSDMD-N (Figure 6(a)). Immunofluorescence results also indicated the colocalization between P62 and GSDMD-N (Figure 6(b)). The protein level of GSDMD-N in the si-ATG5 group was higher than in NP cells treating with si-scr (si-scr group), while the level of GSDMD mRNA was not significantly different between the two groups (Figure 6(c)). Colocalization analysis showed a decreased overlap coefficient in the si-ATG5 group, indicating that autophagy activity could influence the colocalization between P62 and GSDMD-N (Figure 6(d)). To further investigate the degradation mechanism of GSDMD-N, CQ (lysosome pathway inhibitor) and MG132 (proteasome pathway inhibitor) were used in LPS-treated NP cells. Immunoprecipitation and immunofluorescence analysis showed the colocalization between P62 and GSDMD-N in the CQ or MG132 group (Figures 6(e) and 6(f)). CQ-induced lysosome dysfunction resulted in the accumulation of cytoplasmic P62 and decreased the integration between P62 and GSDMD-N, while MG132 showed a nonsignificant effect (Figure 6(g)). These results demonstrated that autophagy regulates GSDMD-N protein levels in P62-mediated selective degradation, which depends on the functional lysosome pathway and not the proteasome pathway.

### 3.6. Administration of VX-765 Retards IDD Progression *In Vivo*

**Figure 7:**
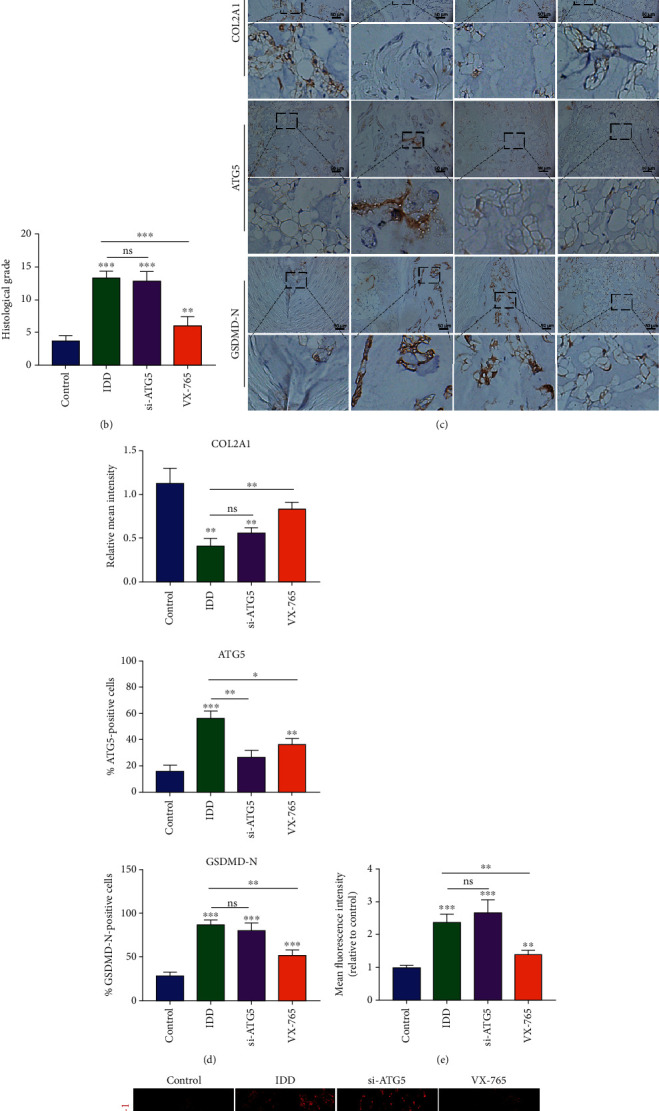
Administration of VX-765 ameliorated the progression of IDD in vivo. (a) Histological staining, including HE, Alcian blue, and Masson staining, showed the morphology of rat intervertebral disc. The IDD, si-ATG5, and VX-765 groups were treated with needle puncture, while the control group is an untreated group. (b) Histological grades were calculated based on the histological staining results. (c) Immunohistochemistry staining for COL2A1, ATG5, and GSDMD-N was conducted to evaluate the expression level of proteins in tissues. (d) Quantification of COL2A1, ATG5, and GSDMD-N in immunohistochemistry staining. (f) Immunofluorescence staining of caspase-1 and (e) quantification results of the caspase-1 mean fluorescence intensity. ^∗∗^*P* < 0.01 and ^∗∗∗^*P* < 0.001 vs. the control group; ns, *P* > 0.05, no significant difference. Data were presented as the means ± SD, *n* = 5.

To further investigate the role of autophagy and pyroptosis in IDD, a rat disc IDD model was designed and conducted by needle puncture. VX-765, a caspase-1 inhibitor, which could efficiently decrease cell pyroptosis, was used in our animal model [27]. Degenerative NP tissues were marked by loss of ECM proteins and replaced by fibrous tissues. Alcian blue and Masson staining showed decreased contents of aggrecan but increased fibrous tissues in the IDD and ATG5 knockdown groups (Figure 7(a)). The histological grade of the VX-765 group was markedly lower than that of the IDD group (Figure 7(b)), indicating a less degenerative profile. Immunohistochemistry results of COL2A1, ATG5, and GSDMD-N revealed that VX-765 treatment decreased GSDMD-N expression, while knockdown of ATG5 promoted the accumulation of GSDMD-N in NP tissues (Figures 7(c) and 7(d)). Immunofluorescence staining also showed that the level of caspase-1 was increased during IDD progression and that VX-765 significantly inhibited caspase-1 expression (Figures 7(e) and 7(f)). These results demonstrated that inhibition of autophagy activity aggravated cell pyroptosis and accelerated the IDD progression, while VX-765 treatment could retard IDD *in vivo*.

## 4. Discussion

**Figure 8:**
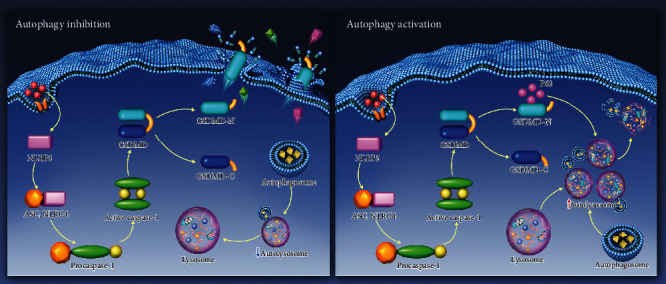
Schematic model illustrating the autophagy regulative mechanism of GSDMD-mediated pyroptosis. Autophagy inhibition promotes the accumulation of cytoplasmic GSDMD-N and elicits cell membrane perforation (a). Upon autophagy activation, P62 mediates the degradation of GSDMD-N in autolysosome and reduces the cell pyroptosis (b). NLRP3: NLR family PYRIN domain-containing 3; ASC: apoptosis-associated speck-like protein containing a CARD; NLRC4: NLR family CARD domain-containing protein 4; GSDMD: gasdermin D; GSDMD-N: N-terminal of gasdermin D; GSDMD-C: C-terminal of gasdermin D; P62: SQSTM1/sequestosome 1.

Various types of programmed cell death contribute to the pathogenesis of IDD, including cell apoptosis, pyroptosis, and necroptosis [28]. In the present study, we revealed that impaired autophagy accelerates the pyroptotic NP cell death induced by LPS. Here, we presented the first evidence that P62-mediated autophagic degradation of the pyroptosis executor, GSDMD-N, serves as a regulatory mechanism of cell pyroptosis. Delivery of VX-765, the inhibitor of caspase-1, inhibits the activation of inflammasome and pyroptosis, thereby ameliorating the IDD progression *in vivo*. Our study suggests the important role of pyroptotic cell death during IDD and investigates the relationship between autophagy and pyroptosis (Figure 8). These results may provide a potential therapeutic target for IDD treatment.

Autophagy is a degrading intracellular mechanism that recycles organelles and proteins to maintain cellular homeostasis [29]. Growing evidence has clarified the protective role of autophagy in IDD development, that activating autophagy could decrease the apoptotic rates of resident cells and promote matrix remodeling during IDD [30, 31]. Consistent with previous studies, our results showed increased expression of autophagy-related protein in degenerated discs [32]. Elevated basal autophagy might adjust resident cells to microenvironmental stress in degenerated discs; however, excessive or inappropriate stimuli could promote autophagic cell death [33]. Autophagy, closely interrelated with apoptosis, is considered as a double-edged sword in cell survival [34]. Indeed, autophagy has a controversial and complicated role in the progression of IDD. Based on the detection of autophagic markers in disc tissues, we found that autophagy levels increased during the progression of IDD. Intervention of autophagy accelerates the death of NP cells, indicating that autophagy is necessary for the survival of disc cells.

Recent researches indicated that pyroptotic death of resident cells in disc promotes the progression of IDD [35, 36]. In senescent or degenerated IVDs, a great deal of inflammatory cytokines or reactive oxygen species (ROS) accumulate, leading to the activation of NLRP3 inflammasome and caspase-1. Activated caspase-1 cleaves GSDMD to release GSDMD-N fragments, which ultimately results in membrane pore formation and cell death [37]. Silencing of GSDMD could inhibit the inflammatory response and reduce oxidative stress, which protect against stimulus-induced organ injury [38]. A previous study also showed that the progression of IDD was effectively retarded after delivering of the NLRP3 inflammasome inhibitor into the degenerated rat disc [35]. Consistent with their results, our study also demonstrated that administration of a caspase-1 inhibitor decreases GSDMD expression and significantly ameliorated IDD *in vivo*. Therefore, the intervention of cell pyroptosis might be an effective therapeutic target in IDD.

Currently, the role of autophagy in pyroptotic cell death is still controversial. Several studies suggested that autophagy inhibits the inflammasome activation and indicated the protective effect of autophagy on cell pyroptosis [36, 39]. A research conducted by Bai et al. revealed that autophagy is activated during ROS-induced cell pyroptosis and autophagy inhibition could aggravate cell pyroptosis [36]. Since autophagosomes could degrade their contents enzymatically, it is reasonable to assume that autophagy activation removes some pyroptotic inducers or clears damaged organelles to reconstruct cellular homeostasis [39]. In the present study, we confirmed a direct connection and colocalization of LC3 and GSDMD-N. Moreover, GSDMD-N could be removed via P62-mediated autophagic degradation. P62 is incorporated into autophagosomes with GSDMD-N and then degraded by lysosomes, resulting in the efficient degradation of GSDMD-N. Other researchers also reported the conspirator role of autophagy in pyroptotic cell death [40, 41]. Wang et al. found that the accumulation of autophagy markers increased the occurrence of pyroptosis which was mainly associated with end-stage autophagy activation and lysosome instability and treatment of autophagy inhibitor reduced the cell pyroptosis [40]. Since autophagy is a diverse-stage activity and involves dynamic processes, a causal link between autophagy and pyroptosis remains unclear and controversial.

Indeed, inflammasome activation, including NLRP3 inflammasome, plays a critical role in pyroptotic cell death [42]. Several studies have indicated that autophagy activation reduces NLRP3 expression and decreases the production of cleaved caspase-1 [43, 44]. Houtman et al. reported a colocalization between NLRP3 and LC3-labeled structures, indicating a selective degradation mechanism of NLRP3 [45]. Besides, the mammalian target of rapamycin (mTOR) signaling pathway is always involved in autophagy activation. Intervention of autophagy could regulate NLRP3 activation via modulating the binding of mTOR and NLRP3 [43]. On the other hand, cell plasma membrane perforation is a symbol of pyroptotic cell death. The perforation of the plasma membrane leads to leakage of cellular contents and release of inflammatory cytokines [46]. Under autophagy activation, the lysosomes could serve as a membrane repair mechanism [47–49]. With a series of small GTPase activation, lysosomes fuse with the plasma membrane and release the degradation products via an exocytosis pathway [47], possibly repairing pyroptotic perforation and promoting cell survival.

Although our study provided enough evidence to elucidate the relationship between autophagy and pyroptosis during IDD progression, there still are several limitations in our research. First, we knock down the ATG5 expression with siRNA transfection, while utilization of ATG-knockout cells or animals may provide firmer evidence. Second, we focused on the autophagy receptor P62/SQSTM1 in the autophagic degradation of GSDMD-N. However, there may be more receptors mediating the autophagy activation and degradation mechanism. Moreover, GSDMD and GSDMD-N are important but not only participants in cell pyroptosis. More pyroptosis-related pathway should be investigated in IDD. Therefore, further research and clinical studies on the role of autophagy and pyroptosis in IDD are required.

In conclusion, our study investigated the relationship between autophagy and cell pyroptosis in human NP cells. Our results clarified the detrimental role of pyroptosis in IDD and the protective role of autophagy in pyroptotic NP cell death. Importantly, our results demonstrated the regulatory role of the autophagy-lysosome pathway in pyroptosis, which may help identify novel therapeutic target for IDD treatment.

## Data Availability

All datasets generated for this study are included in the article.
